# Professionalism and mental health in medical and nursing students under prolonged virtual learning: a longitudinal study in Peru

**DOI:** 10.1186/s12909-026-09750-2

**Published:** 2026-06-17

**Authors:** Martha H Gonzales Sota, Nancy Berduzco-Torres, Montserrat San-Martín, María Gonzalez-Álamos, Luis Vivanco

**Affiliations:** 1 Facultad de Enfermería, Universidad Nacional San Antonio Abad del Cusco, Cusco, 08000 Peru; 2 Departamento de Estadística e Investigación Operativa, Universidad de Granada, Melilla, 52005 Spain; 3 Plataforma de Bioética y Educación Médica, Fundación Rioja Salud (FRS), Logroño, 26006 Spain; 4 Centro Nacional de Documentación en Bioética, Fundación Rioja Salud (FRS), Logroño, 26006 Spain

**Keywords:** Medical professionalism, Depression, Anxiety, Subjective wellbeing, Medicine, Nursing, Virtual learning

## Abstract

**Background:**

After at least one year of virtual learning due to COVID‑19, medical and nursing faculties varied in their return to in‑person training. The absence of in‑person environments may have affected professionalism components such as empathy, teamwork, and lifelong learning abilities, while also impacting students’ mental health and wellbeing. The objective of this study was to measure that effect.

**Methods:**

A longitudinal study was conducted from 2020 to 2023 in the five medical and nursing faculties of Cusco, Peru. Two cohorts were followed: one with prolonged exposure to virtual learning and another with shorter exposure before resuming in‑person activities. Empathy (JSE), teamwork (JSAPNC), lifelong learning (JeffSPLL), depression (PHQ‑9), anxiety (GAD‑7), wellbeing (SWLS), and family loneliness (SELSA) were assessed with validated instruments, together with sociodemographic and academic variables. Analyses included reliability testing, paired and independent group comparisons, and multivariate regression models.

**Results:**

A total of 417 students (233 medicine) completed both assessments; 137 (33%) had prolonged virtual exposure. Wellbeing was positively associated with lifelong learning, age, and shorter virtual exposure, but inversely with empathy and family loneliness. Risk of depression decreased with teamwork and in nursing students but increased in those enrolled in the clinical phase at baseline. Anxiety was associated with greater family loneliness, while a history of severe COVID‑19 illness was associated with lower risk. Students with shorter virtual exposure improved in empathy, teamwork, and learning abilities, whereas those with prolonged exposure did not.

**Conclusions:**

These findings indicate that prolonged reliance on virtual learning undermines professionalism‑related competencies, which are important for students’ mental health and wellbeing. They also highlight the role of family support and the need for medical education to balance technology with in‑person training.

**Supplementary Information:**

The online version contains supplementary material available at 10.1186/s12909-026-09750-2.

## Introduction

Medical professionalism has become a central paradigm in medical education, encompassing technical expertise together with ethical behaviour, communication skills, and a commitment to patient‑centred care [1]. Within this framework, empathy, teamwork, and lifelong learning are widely recognized as essential components of professionalism in medicine and nursing [2]. These competencies have been associated with positive mental health outcomes. In health professionals, they are linked to reduced burnout and better perceived wellbeing [3–5], while in students, they are related to improved academic performance and emotional wellbeing [6, 7].

In 2020, the outbreak of COVID‑19 profoundly disrupted medical education worldwide, forcing universities to replace in‑person training with virtual modalities [8]. Peru was among the countries most severely affected, with one of the highest mortality rates globally. The pandemic exposed serious deficiencies in its healthcare system and led to the adoption of drastic nationwide measures, including prolonged lockdowns and extended reliance on virtual learning [9]. Peruvian medical and nursing faculties were particularly impacted, as students faced reduced opportunities for clinical practice and social interaction, conditions that may have hindered the acquisition of professionalism‑related competencies and affected their mental health [10, 11], contributing to increased stress levels and higher dropout rates among these students [6, 12].

Evidence suggests that prolonged reliance on virtual learning limits opportunities for mentorship and direct patient contact and may hinder the development of interpersonal and collaborative abilities [13]. This effect is particularly detrimental in health professions students [9], whose profiles are often oriented toward social interaction and patient‑centred activities, in contrast with other disciplines where technical competencies predominate and the absence of in‑person contact may be less disruptive. Beyond the effects of virtual learning, medical education has often placed greater emphasis on technical aspects than on the cultivation of social abilities [14]. This imbalance negatively affects the training of future physicians and is reinforced by the way these competencies are commonly labelled as “hard” and “soft” skills, a terminology that conveys the misleading idea that technical abilities are more robust or valuable than interpersonal ones [15]. Such concerns are not limited to medicine but extend broadly across health professions [16].

The lack of in‑person social interactions in virtual learning environments may also be detrimental to students’ mental health and emotional wellbeing [17]. These concerns are particularly relevant when virtual learning is not complemented by in‑person learning activities [18]. In health care professions, it is essential that students acquire, from the earliest stages of their training, competencies such as empathy, teamwork, and learning abilities, which are best developed in environments that provide direct opportunities for patient contact, collaboration, and experiential learning. This situation changed drastically during the pandemic [19], when schools were required to adapt their programs to virtual learning environments. While some institutions introduced these changes temporarily, others adopted them permanently.

From a conceptual perspective, professionalism-related competencies such as empathy, teamwork, and lifelong learning can be understood as psychosocial and behavioral resources that support students’ mental health and wellbeing. These competencies are closely linked to mental health outcomes, although their effects may vary depending on context and individual factors. They contribute to emotional regulation, adaptive coping, and the ability to establish meaningful interpersonal relationships, which are essential in clinical practice [4]. In educational environments, evidence suggests that contextual factors, including both educational environments [20] and family support [21], may play an important role in coping with psychological distress and in shaping the early development of professionalism-related competencies. Particularly, family loneliness appears as a key contextual factor that may directly affect mental health outcomes and interact with the development of professionalism-related competencies, especially those related to social and interpersonal abilities such as empathy [21], as well as self-regulatory and motivational capacities such as lifelong learning [7], thereby shaping their overall impact on students’ wellbeing.

This framework provides the basis for examining how educational conditions may influence both the development of these competencies and their relationship with mental health outcomes.

### Study purpose

On this basis, the present study was designed to measure the impact of curricular decisions on the development of medical professionalism and on students’ mental health and subjective wellbeing. Two cohorts of medical and nursing students were followed prospectively for three years (2020–2023): one with prolonged exposure to fully virtual learning, and another with shorter exposure through programs combining virtual and in‑person learning activities. It was hypothesized that greater medical professionalism, operationalized through validated measures of empathy, teamwork, and lifelong learning, would be associated with a lower risk of depression and anxiety and with higher subjective wellbeing at the end of the study period, even after accounting for sociodemographic and academic factors. In addition, it was hypothesized that prolonged exposure to fully virtual learning would negatively affect the development of these abilities, such that students in the prolonged‑virtual cohort would show smaller improvements over time compared to those in the shorter‑virtual cohort.

## Methods

### Participants and procedures

A longitudinal survey‑based study was conducted in the two medical and three nursing faculties located in Cusco, Peru, from May 2020 to May 2023. Undergraduate medical and nursing students participated in two waves of data collection: the first in May 2020 and the second in May 2023. Students were informed about the study in sessions led by members of the research team (NB and MG), virtually in the first wave and in person in the second, with emphasis on voluntary participation, confidentiality, and independence from academic evaluation. Those who agreed received an email with the electronic informed consent form, which they signed before completing a questionnaire that included psychometric measures and sociodemographic data.

Students who did not participate in both waves, resided abroad, or failed to complete at least one scale in both waves were excluded. The study was conducted in collaboration between institutions in Peru and Spain, in accordance with the Declaration of Helsinki and national regulations. Ethical approval was obtained from the Research Ethics Committee of La Rioja, Spain (CEImLAR‑PI‑440), and the Research Ethics Committee of the National University of the Altiplano, Peru (03‑CIEI‑UNA‑PUNO), both acting as independent ethics committees. The Jefferson Scale of Empathy was administered with written permission from Dr. Mohammadreza Hojat, the SELSA with authorization from Dr. DiTommaso, the PHQ‑9 and GAD‑7 with permission from Professor Kurt Kroenke, and the SWLS with proper attribution to Ed Diener and colleagues.

### Medical professionalism

#### Clinical empathy

Two versions of the 20‑item Jefferson Scale of Empathy (JSE) were used to assess students’ orientation toward empathy in clinical encounters: the JSE‑S for medical students and the JSE‑HPS for nursing students [22, 23]. Items are rated on a 7‑point Likert scale, from strongly disagree (1) to strongly agree (7). The two versions differ only in wording, replacing “physician/medicine” with terms appropriate for other health disciplines. For example, the JSE‑S item “Physicians should try to stand in their patients’ shoes when providing care” is rephrased in the JSE‑HPS as “Health care providers should try to stand in their patients’ shoes when providing care.” Scores range from 20 to 140, with higher scores indicating greater empathetic orientation.

#### Teamwork abilities

The 15‑item Jefferson Scale of Attitudes toward Physician–Nurse Collaboration (JSAPNC) was used to assess teamwork abilities between physicians and nurses [24]. These abilities refer to attributes required for working cooperatively, sharing responsibilities in problem‑solving, and making joint decisions in patient care. Items are rated on a 4‑point Likert scale, from strongly disagree (1) to strongly agree (4). Scores range from 15 to 60, with higher scores indicating greater development of teamwork abilities.

#### Lifelong learning abilities

Two versions of the 14‑item Jefferson Scale of Lifelong Learning (JeffSPLL) were used to assess lifelong learning abilities [25, 26]. These abilities involve skills related to information gathering, use of learning opportunities, and self‑motivation in professional studies. The version for medical students (JeffSPLL‑MS) and the version for nursing students (JeffSPLL‑HPS) differ only in wording, replacing “medicine/physician” with terms appropriate for other health disciplines. Items are rated on a 4‑point Likert scale, from strongly disagree (1) to strongly agree (4). Scores range from 14 to 56, with higher scores indicating greater development of lifelong learning abilities.

### Mental health and subjective wellbeing

#### Depression symptoms

The 9‑item Patient Health Questionnaire (PHQ‑9) was used to assess depressive symptoms over the last two weeks, with items scored 0–3 and total scores ranging 0–27 [27]. In line with Peruvian validation studies [28, 29], participants scoring ≥ 7 were classified as presenting depressive symptoms, a threshold adopted in the absence of systematic clinical interview validation. For international comparability, a cut‑off of ≥ 10 was also reported [27].

#### Anxiety symptoms

The 7‑item Generalized Anxiety Disorder Scale (GAD‑7) was used to assess anxiety symptoms over the last two weeks, with items scored 0–3 and total scores ranging 0–21 [30]. Participants scoring ≥ 8 were classified as presenting anxiety symptoms, a cut‑off supported by validation in Peruvian hospital patients [28] and in Peruvian university students [31]. This threshold was adopted given the absence of clinical interview validation, while a cut‑off of ≥ 10 was also reported to facilitate international comparability.

#### Subjective wellbeing

The 5‑item Satisfaction With Life Scale (SWLS), Spanish version, was used to assess subjective well‑being [32]. Items are rated on a 5‑point Likert scale, from strongly disagree (1) to strongly agree (5). Higher scores indicate greater levels of subjective well‑being.

### Teaching modality

Teaching modality was classified as “virtual” (online only, without laboratory or clinical training), “hybrid” (online plus in‑person activities with some restrictions), or “in‑person” (full on‑site teaching, with minor adjustments). All faculties adopted virtual mode in April 2020 due to COVID‑19 restrictions. By 2023, all had resumed in‑person teaching, though their trajectories differed. For analytic purposes, this variable was reduced to two categories: “prolonged virtual exposure” (students from faculties that remained two years in virtual mode before transitioning) and “shorter virtual exposure” (students from faculties that experienced only one year in virtual mode before returning earlier to in‑person teaching).

### Other variables collected

Two sets of variables were collected during the study, at baseline and at follow‑up.

#### Baseline

Sex (male, female), age, university, discipline (medicine, nursing), and academic year of enrolment were recorded. Students also indicated whether their career choice was a personal decision (“vocation‑driven” group) or was influenced by external factors such as relatives, economic circumstances, or other considerations (“externally influenced” group). In addition, family loneliness was assessed using the family domain of the Social and Emotional Loneliness Scale for Adults (SELSA‑S) [33]. This subscale consists of 5 items developed according to Weiss’s typology of loneliness, rated on a 7‑point Likert scale from strongly disagree (1) to strongly agree (7). Higher scores indicate greater perceived loneliness in the family context, which can be interpreted as an inverse indicator of perceived family support. One derived variable was constructed from year of enrolment and discipline: “training stage”, which classified medical students in their first two years and nursing students in their first year as “pre‑clinical”, with all others as “clinical”.

#### Follow‑up

At the end of the study, students were asked whether their perception of their career choice had changed over time, responding to a multiple‑choice question with three possible options (“it is better”, “it is the same”, “it is worse”). They also indicated whether at any point they had considered dropping out of their studies (“never”, “at least once”). Academic progression was defined by subtracting the academic year recorded at baseline from that reached at follow‑up; students advancing three years were categorized as having “adequate progression”, while those advancing fewer than three years were categorized as “inadequate progression”. COVID‑19 illness severity was retrospectively classified into four levels: no infection/asymptomatic, mild with ambulatory care, mild with hospital care, and severe, based on the most severe episode reported.

### Data analysis

A descriptive analysis was performed for the whole sample and separately for the two study cohorts. The distribution of categorical variables, such as vocational choice, intention to drop out, teaching modality, and career perception, was compared between cohorts using chi‑squared tests to assess comparability. Since sex is unbalanced in both disciplines, particularly in nursing, this variable was not analysed. Instead, discipline (medicine vs. nursing) was used as a covariate, as it provided a more balanced and analytically meaningful distinction.

Only fully completed scales were included in the statistical analysis. The reliability of each scale was assessed by calculating Cronbach’s alpha and McDonald’s omega coefficients. Normality was examined using Pearson’s chi‑squared and Lilliefors‑Kolmogorov‑Smirnov tests, and since score distributions did not follow normality, non‑parametric tests were applied in subsequent analyses.

To address the first hypothesis, subjective wellbeing (measured by the SWLS) at the end of the study was analysed through multiple linear regression in an ANCOVA framework, with baseline wellbeing included as a covariate. Empathy, teamwork, lifelong learning, and family loneliness scores were entered as explanatory variables, together with sociodemographic and academic covariates such as age, discipline, training stage, academic progression, vocational choice, intention to drop out, and final perception of the profession. For analytic purposes, the variable career perception was dichotomized into “better” and “equal or worse.” A model was accepted only when it met the necessary conditions for statistical inference: normality, zero mean, constant variance and uncorrelatedness of the residuals, in addition to linearity and absence of multi‑collinearity. Depression (measured by the PHQ‑9) and anxiety (measured by the GAD‑7) were examined separately using multiple logistic regression models with the same predictors and covariates. Effect sizes were calculated in all cases.

To address the second hypothesis, the impact of prolonged exposure to fully virtual learning on the development of competencies was examined across and within cohorts. First, baseline differences between cohorts in empathy, teamwork, and lifelong learning abilities were assessed using Mann–Whitney tests to establish initial comparability, ensuring that observed differences in change were not driven by initial imbalances. Then, within each cohort, paired Wilcoxon tests were applied to compare baseline and final scores in these competencies. Finally, changes in scores were compared between cohorts using Mann–Whitney tests to corroborate differences in competency development attributable to teaching modality. Effect sizes were calculated for all comparisons.

All analyses were done in R language and programming environment for statistical and graphical analysis, RStudio version 2024 for Windows, and with the statistical analysis packages nortest, multilevel, fmsb, lsr, rstatix, psych, dplyr, and rstatix.

## Results

### Preliminary findings

At baseline in 2020, 787 students participated (507 female), of whom 417 (296 female) also completed assessments in 2023. Among these, 138 (33%) experienced prolonged virtual exposure and 279 (67%) shorter exposure. No baseline differences were observed between groups in sociodemographic and academic variables, except for training stage (*p* = 0.03), with more preclinical students in the prolonged‑virtual group (Table 1).

**Table 1:** Baseline sociodemographic and academic variables by teaching modality

	Entire sample	Virtual modality		*p*-value
		Prolonged	Shorter	
*n*	417	138 (33%)	279 (67%)	--
Sex				0.50^a^
Male	121 (29%)	43 (32%)	78 (28%)	
Female	296 (71%)	95 (68%)	201 (72%)	
Age				0.34^b^
M (*SD*)	20 (3)	20 (2)	20 (4)	
Mdn [Min–Max]	20 [17–49]	19 [17–33]	20 [17–49]	
Discipline				0.83^a^
Medicine	278 (67%)	91 (66%)	187 (67%)	
Nursing	139 (33%)	47 (34%)	92 (33%)	
Training stage				0.03^a^
Pre-clinical stage	229 (55%)	86 (62%)	143 (51%)	
Clinical stage	188 (45%)	52 (38%)	136 (49%)	
Career choice				0.95^a^
Vocation-driven	297 (71%)	98 (71%)	199 (71%)	
Externally influenced	120 (29%)	40 (29%)	80 (29%)	
Family loneliness (SELSA-S/f)				0.06^b^
M (*SD*)	12(7)	11(6)	13(8)	
Mdn [Min–Max]	10 [5–35]	9 [5–35]	11 [5–35]	

^a^Chi-square test

^b^Mann-Whitney U test

All scales showed good to excellent reliability (Cronbach’s alpha 0.81–0.92; McDonald’s omega 0.87–0.94). Descriptive statistics and reliability indices are summarized in Table 2.

**Table 2:** Descriptive analysis and reliability of scales administered

	*n*	PR	AR	Mdn	M (SD)	Alpha	Omega
Baseline (2020)							
Professional competencies							
Empathy (JSE)	405	20–140	33–136	105	103 (18)	0.86	0.90
Teamwork (JSAPNC)	404	15–60	15–60	47	46 (8)	0.87	0.90
Learning (JeffSPLL)	410	14–56	17–56	45	44 (7)	0.85	0.90
Subjective wellbeing (SWLS)	410	5–25	5–25	18	18 (4)	0.84	0.87
Family loneliness (SELSA-S/f)	411	5–35	5–35	10	12 (7)	0.85	0.88
Follow-up (2023)							
Professional competencies							
Empathy (JSE)	417	20–140	75–139	108	107 (15)	0.81	0.87
Teamwork (JSAPNC)	416	15–60	24–60	48	48 (7)	0.85	0.89
Learning (JeffSPLL)	417	14–56	28–56	44	45 (8)	0.84	0.88
Subjective wellbeing (SWLS)	410	5–25	5–25	17	17 (5)	0.87	0.90
Family loneliness (SELSA-S/f)	416	5–35	5–35	11	13 (7)	0.85	0.91
Mental health							
Depression (PHQ-9)	416	0–27	0–27	10	11 (6)	0.91	0.93
Anxiety (GDS-7)	417	0–21	0–21	10	11 (5)	0.92	0.94

At follow‑up, academic progression, dropout intention, career perception, COVID‑19 illness severity, and family loneliness were collected. No differences were found for career perception (*p* = 0.35), COVID‑19 illness severity (*p* = 0.37), or family loneliness (*p* = 0.90). Significant differences were observed for academic progression and dropout intention (Table 3).

**Table 3:** Follow-up outcome variables by teaching modality

	Entire sample	Virtual modality		*p*-value
		Prolonged	Shorter	
*n*	417	138 (33%)	279 (67%)	
Academic progression				< 0.001^a^
Adequate	292 (70%)	116 (84%)	176 (43%)	
Inadequate	125 (30%)	22 (16%)	103 (37%)	
Dropout intention				0.003^a^
Never	192 (46%)	78 (57%)	114 (41%)	
At least once	225 (54%)	60 (43%)	165 (59%)	
Career perception				0.35^a^
Better	149 (36%)	45 (33%)	104 (37%)	
Same or worse	268 (64%)	93 (67%)	175 (63%)	
COVID-19 infection severity				0.37^b^
No infection/Asymptomatic	85 (20%)	25 (18%)	60 (21%)	
Mild	173 (41%)	57 (41%)	116 (42%)	
Mild-severe	13 (3%)	2 (1%)	11 (4%)	
Severe	146 (36%)	54 (40%)	92 (33%)	
Family loneliness (SELSA-S/f)				0.90^c^
M (*SD*)	13(7)	12(6)	13(7)	
Mdn [Min–Max]	11[5–35]	11[5–35]	11[5–35]	

^a^Chi-square test

^b^Fisher’s exact test

^c^Mann-Whitney U test

### Main findings

#### Subjective wellbeing

A regression model was obtained explaining 32.4% of the variance in subjective wellbeing (R²-adjusted = 0.312; *F*_(7,389)_ = 26.66; *p* < 0.001). Increases in lifelong learning abilities (*p* = 0.002) were positively associated with wellbeing. In contrast, increases in empathy (*p* < 0.001) and higher levels of family loneliness, both at baseline (*p* < 0.001) and as increases during the study period (*p* < 0.001), were negatively associated with wellbeing. Student age (*p* = 0.006) and being enrolled in programs with shorter virtual exposure (*p* = 0.010) were also associated with higher wellbeing (Table 4).

**Table 4:** Multiple regression model for subjective wellbeing at the follow-up measurement

	β	SE	t	*p*-value
Change in empathy (follow-up – baseline)	-0.04	0.01	-3.63	< 0.001
Lifelong learning abilities (baseline)	+ 0.14	0.04	+ 3.15	0.002
Lifelong learning abilities (follow-up – baseline)	+ 0.16	0.04	+ 4.38	< 0.001
Family loneliness (baseline)	-0.29	0.04	-7.60	< 0.001
Change in family loneliness (follow-up – baseline)	-0.32	0.03	-10.67	< 0.001
Age	+ 0.16	0.06	+ 2.77	0.006
Shorter virtual exposure	+ 1.11	0.43	+ 2.57	0.010

β beta coefficient, *SE* standard error, *t *t-value, *p p*-value

#### Depression

A logistic regression model was obtained using the Peruvian cut‑off (≥ 7). Baseline teamwork was protective (*p* = 0.019), while family loneliness, both at baseline (*p* < 0.001) and as increases over time (*p* < 0.001), was consistently associated with higher risk of depression. Nursing students (*p* < 0.001) and those in advanced academic years (*p* < 0.001) showed lower risk, whereas being in the clinical training stage at baseline was strongly associated with higher risk (*p* < 0.001). The model demonstrated moderate explanatory power (Nagelkerke R²=0.23). In the model using the international cut‑off (≥ 10), the same set of predictors was maintained, with an explanatory power of Nagelkerke R²=0.197. The detailed results are presented in Table 5 for the Peruvian cut‑off, and in Supplementary Table 1 for the international cut‑off.

**Table 5:** Logistic regression models for depression and anxiety (Peruvian cut-offs ≥ 7 and ≥ 8)

	β	SE	OR (95% CI)	*p*-value
Depression				
Teamwork (baseline)	-0.05	0.02	0.95 (0.92–0.99)	0.019
Family loneliness (baseline)	+ 0.13	0.03	1.14 (1.08–1.22)	< 0.001
Change in family loneliness (follow-up – baseline)	+ 0.14	0.03	1.15 (1.10–1.21)	< 0.001
Discipline: nursing	-2.30	0.62	0.10 (0.028–0.32)	< 0.001
Academic course (baseline)	-2.15	0.57	0.12 (0.035–0.33)	< 0.001
Training stage: clinical phase	+ 2.31	0.69	10.1 (2.76–41.4)	< 0.001
Anxiety				
Family loneliness (baseline)	+ 0.13	0.02	1.14 (1.09–1.20)	< 0.001
Change in family loneliness (follow-up – baseline)	+ 0.11	0.02	1.12 (1.07–1.16)	< 0.001
Self-reported COVID-19 illness severity	-0.19	0.09	0.83 (0.68–0.99)	0.045

β logistic regression coefficient, *SE* standard error, *CI* confidence interval, *OR* Odds ratio, *p p*-value

#### Anxiety

A logistic regression model was obtained identifying three predictors: family loneliness at baseline (*p* < 0.001), increases in family loneliness (*p* < 0.001), and COVID‑19 illness severity (*p* = 0.045). The first two were associated with higher risk of anxiety, while greater illness severity was linked to lower risk. The model showed moderate explanatory power (Nagelkerke R²=0.146). In the model using the international cut‑off (≥ 10), the same predictors were maintained, with an explanatory power of Nagelkerke R²=0.147. The detailed results are presented in Table 5 for the Peruvian cut‑off and in Supplementary Table 1 for the international cut‑off.

#### Development of medical professionalism competencies

**Fig. 1:**
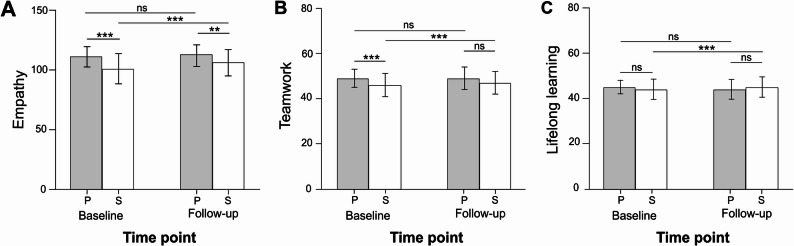
Differences in empathy (**A**), teamwork (**B**), and lifelong learning (**C**) across cohorts exposed to prolonged (P) versus shorter (S) virtual exposure. ***p < 0.001, **p < 0.01

At baseline, the prolonged‑virtual cohort scored higher in empathy (*p* < 0.001, *r* = 0.34) and teamwork (*p* < 0.001, *r* = 0.21), but not in lifelong learning abilities (*p* = 0.080). At follow‑up, this cohort showed no significant changes in any professional competency. In contrast, the shorter‑virtual cohort improved in empathy (*p* < 0.001, *r* = 0.30), teamwork (*p* < 0.001, *r* = 0.29), and lifelong learning abilities (*p* = 0.0017, *r* = 0.19). Empathy remained higher in the prolonged‑virtual cohort in comparison with the shorter one (*p* = 0.009, *r* = 0.13), while differences in teamwork (*p* = 0.23) and lifelong learning abilities (*p* = 0.24) were not significant. Findings are summarized in Fig. 1.

## Discussion

### Subjective wellbeing

The multiple linear regression model highlights the interplay between psychosocial and academic factors in wellbeing. Lifelong learning abilities were positively associated with wellbeing, both at baseline and as improvements over time, reinforcing their role in resilience and adaptation. Age and reduced exposure to virtual learning were also positively associated with wellbeing, suggesting that maturity and balanced training environments contribute positively. In contrast, family loneliness remained a strong negative determinant, consistent with evidence that lack of family support exacerbates vulnerability [7, 21, 34]. Importantly, increases in empathy were negatively associated with wellbeing, perhaps reflecting heightened awareness of unmet formative needs under virtual conditions [18, 35]. This apparent contradiction may be better understood by distinguishing cognitive or clinical empathy from emotional empathy (also called sympathy). As measured by the Jefferson Scale of Empathy, empathy in this study refers primarily to the former, a predominantly cognitive attribute involving understanding rather than feeling patients’ experiences, together with the capacity to communicate that understanding and an intention to help [22]. By contrast, emotional empathy or sympathy refers to a predominantly affective reaction to another person’s suffering, which, when overabundant, may become overwhelming and contribute to emotional exhaustion [36]. In the context of prolonged virtual learning and reduced opportunities for direct patient contact, greater empathy may therefore coexist with poorer wellbeing if the cognitive disposition to understand others is not accompanied by sufficient experiential support and emotional regulation. These findings align with prior studies on family support [7, 21, 37, 38], international evidence on clinical empathy [14], and the impact of quarantine on students’ distress [39]. Together, they underscore the need to strengthen lifelong learning, mitigate family loneliness, and safeguard personal development and clinical skills.

### Depression

In depression, teamwork acted as a protective factor, underscoring the value of collaborative environments [3, 5, 40]. Conversely, family loneliness both at baseline and over time was consistently linked to higher risk, confirming its central role in student vulnerability [7, 21, 34]. Beyond psychosocial determinants, the academic context contributed significantly: both being in advanced academic years and studying nursing were associated with lower risk of depression. This may reflect, on the one hand, the experience and maturity gained in higher courses, and on the other, characteristics linked to the nursing profile and professional role, such as emotional regulation, resilience, and coping abilities, plausibly mitigating depressive risk [41–43]. Yet COVID‑19 restrictions on patient contact limited experiential learning, which may explain why students in clinical training reported higher depressive symptoms, a paradox that highlights the tension between professional identity formation and disrupted practice [44].

### Anxiety

For anxiety, family loneliness again emerged as a recurrent determinant, mirroring depression and reinforcing the critical importance of social support [7, 21, 34, 38]. Interestingly, greater severity of COVID‑19 illness was linked to lower anxiety, possibly reflecting a recalibration of risk perception among students who experienced severe episodes and re‑evaluated subsequent threats.

### Impact of virtual learning on professionalism

The findings indicate that virtual education conditions affected the development of medical professionalism competencies. Literature warns that privileging technical knowledge over interpersonal contact may undermine professional identity [15, 18, 45, 46]. Consistent with this, students with prolonged virtual exposure reported lower preparedness in clinical and relational skills, while those with more in‑person training showed better outcomes. This aligns with evidence on the limitations of digital learning in nursing [45] and global challenges post‑COVID‑19 [46]. Taken together, these results reinforce the need for institutional strategies that integrate digital resources with structured opportunities for experiential practice and personal development. While these findings must be interpreted considering the specific context of the COVID‑19 pandemic in Peru, including prolonged lockdowns and extended reliance on virtual learning, the underlying relationships observed may be relevant to other health professions education settings. In particular, the associations between professionalism-related competencies, educational conditions, and mental health outcomes are consistent with previous research and may be applicable in contexts where similar disruptions in clinical training or shifts toward virtual education occur [47]. However, the magnitude of these effects may vary depending on local socioeconomic conditions, institutional resources, and the structure of educational programs.

### Limitations and strengths

This study has limitations that should be acknowledged. Findings reflect a specific regional context, namely Peruvian medical and nursing education during the COVID‑19 pandemic, including prolonged restrictions and reliance on virtual learning, which may limit the direct generalizability of the results to other educational settings with different structural or social conditions. Sex was not analysed due to imbalance across disciplines, with discipline used as a covariate. Career perception was dichotomized, potentially reducing sensitivity. Reliance on self‑report instruments and non‑parametric tests introduces constraints, and exposure to virtual learning was determined by curricular decisions, not experimental assignment.

Despite these limitations, strengths include comprehensive regional coverage across all faculties, a longitudinal design over three years, use of internationally validated instruments, and rigorous analytic procedures. Importantly, the study captures a unique historical moment in medical education, adding contextual value to the findings.

## Conclusions

Taken together, these findings indicate that pandemic restrictions limited experiential learning, and that prolonged virtual learning carried a cost in the development of competencies essential to medical professionalism. This effect was both academic and psychological, as reduced patient contact reinforced feelings of insufficient preparation and contributed to higher depressive symptoms. At the same time, psychosocial and academic variables jointly shaped mental health, with accumulated experience and the nursing profile mitigating risk. These results underscore the need for institutional strategies that safeguard clinical training while addressing psychological consequences through teamwork, social support, and targeted interventions.

## Supplementary Information

Supplementary Material 1.

## Data Availability

The datasets used and analysed during this study are available from the corresponding author on reasonable request.
