# Supplementary material for: Professionalism and mental health in medical and nursing students under prolonged virtual learning: a longitudinal study in Peru
Source: BMC Med Educ. 2026 Jun 17;26:1223. doi: 10.1186/s12909-026-09750-2 (PMC13421740; doi:10.1186/s12909-026-09750-2)
Supplement: Supplementary file 1 — Supplementary Material 1. [file 12909_2026_9750_MOESM1_ESM.pdf]

Supplementary Table 1. Logistic regression models for depression and anxiety (International cut-off ≥10)

	**β**	***SE***	**OR (95% CI)**	***p*-value**
**Depression**				
Teamwork (baseline)	-0.010	0.02	0.99 (0.96–1.02)	0.49
Family loneliness (baseline)	+0.11	0.02	1.12 (1.07–1.17)	<0.001
Change in family loneliness (follow-up – baseline)	+0.12	0.02	1.13 (1.09–1.17)	<0.001
Discipline: nursing	-1.33	0.42	0.27 (0.11–0.59)	0.0015
Academic course (baseline)	-0.98	0.38	0.37 (0.17–0.77)	0.009
Training stage: clinical phase	+1.05	0.50	2.85 (1.08–7.73)	0.036
**Anxiety**				
Family loneliness (baseline)	+0.11	0.02	1.11 (1.07–1.16)	<0.001
Change in family loneliness (follow-up – baseline)	+0.11	0.02	1.11 (1.08–1.15)	<0.001
Self-reported COVID-19 illness severity	-0.19	0.09	0.83 (0.69–0.99)	0.039

β, logistic regression coefficient; *SE*, standard error; CI, confidence interval; OR, Odds ratio; *p*, p-Value
